# Macular Telangiectasia Type 2: A Case Series

**DOI:** 10.7759/cureus.115782

**Published:** 2026-09-04

**Authors:** Vinith R Devarakonda, Gurunadh S Velamakanni, Padala V Prasad, Kandula Satish, Gattineni Dharani

**Affiliations:** 1 Ophthalmology, GSL Medical College and General Hospital, Rajahmundry, IND

**Keywords:** idiopathic macular telangiectasia, macular neurodegeneration, macular telangiectasia type 2, multimodal imaging, oct (optical coherence tomography), retinal telangiectasia

## Abstract

Macular telangiectasia (MacTel) type 2, or juxtafoveal telangiectasia, refers to several vascular abnormalities affecting the capillaries of the foveal and perifoveal regions. It is a progressively degenerative disorder that reduces quality of life by impairing both distance and near vision.

This report pertains to a series of five cases of MacTel type 2 that presented to our Outpatient Department. The patients, in their 50s, presented with metamorphopsia and reduced reading skills. The diagnosis was determined through bilateral anomalous macular presentations, asymmetric foveal pit configuration, disruptions in the inner segment/outer segment layer, and hyperreflectivity and shadowing noted at the fovea on optical coherence tomography (OCT). This series highlights the spectrum of clinical and OCT manifestations and underscores the importance of early detection to prevent misinterpretation and unnecessary interventions.

## Introduction

Macular telangiectasia (MacTel) is a macular disease characterized by abnormalities of the capillaries of the foveal or perifoveal region. MacTel is divided into three main types. Type 1 is congenital, unilateral, uncommon, and thought to be a variant of Coats disease. Type 2 MacTel is the most common type, is bilateral, and is acquired; it is usually found in middle-aged or older patients, with a slight female preponderance of 58%. Type 3 is a rare and poorly understood entity [1,2].

Chronic neurodegenerative processes, vascular inflammation, occlusion, and ectatic capillary changes lead to loss of the outer retinal layers and formation of pseudolamellar macular holes with cystic-like cavitations that can also be seen in both the outer and inner retinal layers [3,4]. As the disease progresses, retinal pigment epithelium (RPE) hyperplasia and migration into the inner retinal layers are seen. Patients can develop retinal neovascularization, which can cause exudation, intraretinal and subretinal hemorrhages, and disciform scarring.

The MacTel Project found that 28% of patients with type 2 MacTel had concomitant diabetes mellitus (DM) and 52% had concomitant hypertension (HTN), hinting at long-term vascular stress [5].

MacTel is primarily neurodegenerative, with vascular remodeling occurring as a consequence, thereby explaining the lack of meaningful response to anti-vascular endothelial growth factor (anti-VEGF) agents. However, treatment options still include anti-VEGF agents in cases of neovascularization [1,2].

This case series describes the clinical and optical coherence tomography (OCT) features of MacTel type 2 in patients without diabetes or HTN.

## Case presentation

Five patients diagnosed with MacTel type 2 were identified among patients presenting to our ophthalmology clinic between December 2023 and August 2025. All patients underwent comprehensive ophthalmic evaluation, including best-corrected visual acuity (BCVA), fundus examination, color fundus photography using the VISUCAM 500 fundus camera (Carl Zeiss Meditec AG, Jena, Germany), and OCT using the PRIMUS 200 OCT system (Carl Zeiss Suzhou Co., Ltd., Suzhou, China). The demographic data, visual acuity (VA), fundus findings, and OCT findings of the five patients are summarized in Table 1.

**Table 1:** Demographic, visual acuity, fundus, and OCT findings in five patients with macular telangiectasia type 2 BCVA: best-corrected visual acuity; OCT: optical coherence tomography

S. No.	Age	Sex	Co-morbidities	BCVA	Fundus Picture	OCT
1	56	F	None	6/36; 6/24	Bilateral subtle parafoveal retinal graying with alteration/loss of the normal foveal reflex and pigmentary changes	Hyperreflectivity in outer layers with shadowing in both eyes
2	57	M	None	6/24; 6/9	Bilateral subtle parafoveal retinal graying with alteration of the foveal reflex.	Hyperreflectivity in outer layers with shadowing in the right eye and inner retinal layer cavitation in the left eye
3	52	F	None	6/24; 6/24	Bilateral subtle parafoveal retinal graying with loss of normal foveal reflex.	Hyperreflectivity in outer layers with shadowing in both eyes
4	58	F	None	6/18; 6/36	Bilateral parafoveal retinal graying with alteration of the foveal reflex	Hyperreflectivity in outer layers with shadowing in both eyes
5	55	F	None	6/36; 6/18	Bilateral subtle parafoveal retinal graying with loss of normal foveal reflex	Loss of IS/OS differentiation in both eyes

Case A

A 56-year-old female patient with no known comorbidities presented with a BCVA of 6/36 OD and 6/24 OS. On fundus examination, there was bilateral subtle parafoveal retinal graying, with alteration/loss of the normal foveal reflex and pigmentary changes. OCT showed hyperreflectivity of the outer retinal layers, with shadowing in both eyes. Figure 1 shows the corresponding fundus and OCT findings.

**Figure 1:**
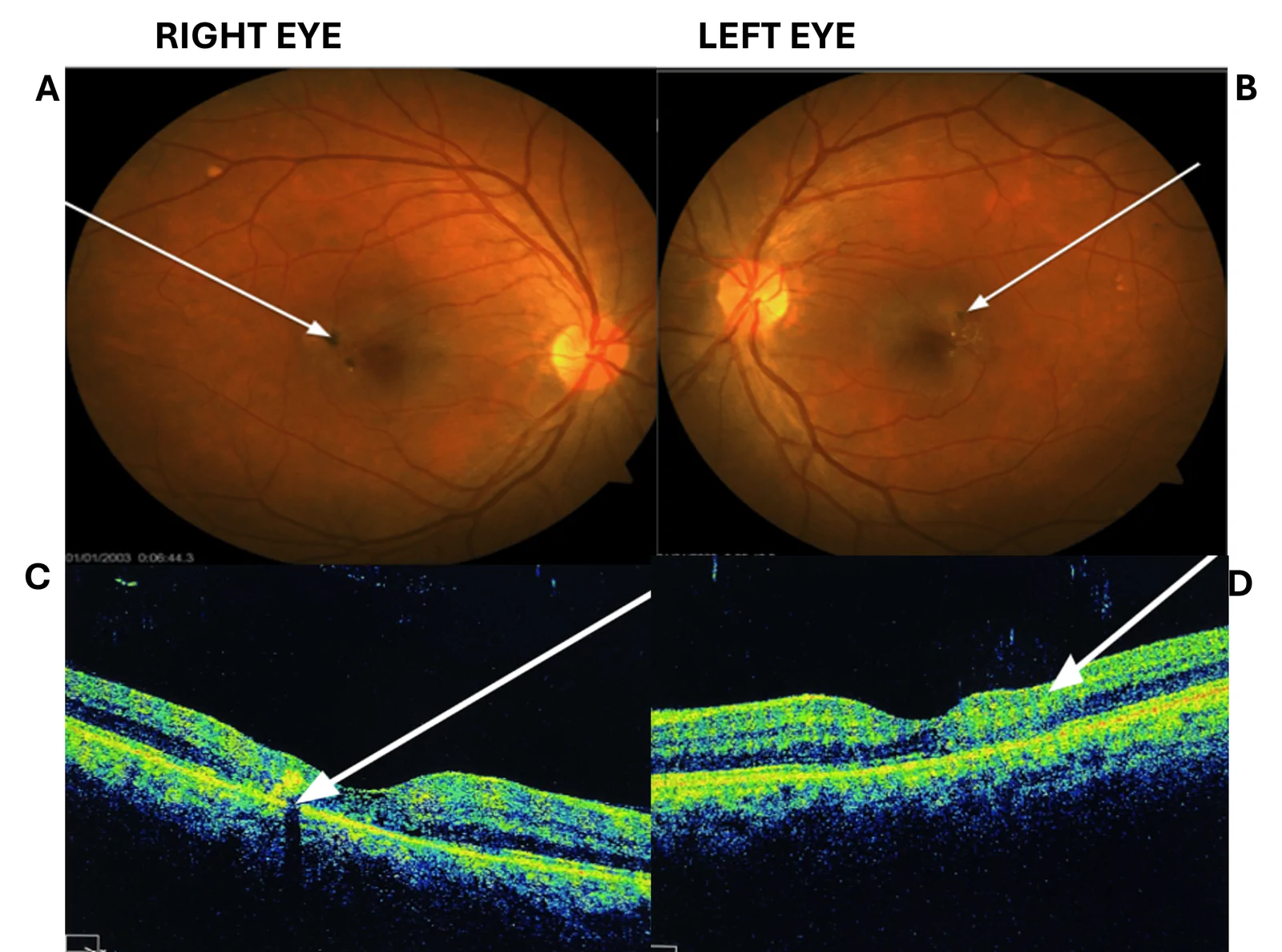
Fundus and optical coherence tomography (OCT) of Case A Fundus photograph of the right eye (A) reveals mild parafoveal retinal graying with disruption of the usual foveal reflex (arrow). (B) Left-eye fundus image demonstrating parafoveal retinal graying with loss of the typical foveal reflex (arrow). (C) Right-eye OCT demonstrating loss of differentiation of the ellipsoid zone (IS/OS zone) at the fovea (arrow). (D) Left-eye OCT demonstrating hyperreflectivity of the outer retinal layers with posterior shadowing at the fovea (arrow).

Case B

A 57-year-old male with no known comorbidities presented with a BCVA of 6/24 in the right eye and 6/9 in the left eye. Fundus examination revealed bilateral subtle parafoveal retinal graying with alteration of the normal foveal reflex. OCT showed hyperreflectivity in the outer retinal layers with shadowing in the right eye, while the left eye showed cavitation due to loss of differentiation of the IS/OS zone. Figure 2 shows the corresponding fundus and OCT findings.

**Figure 2:**
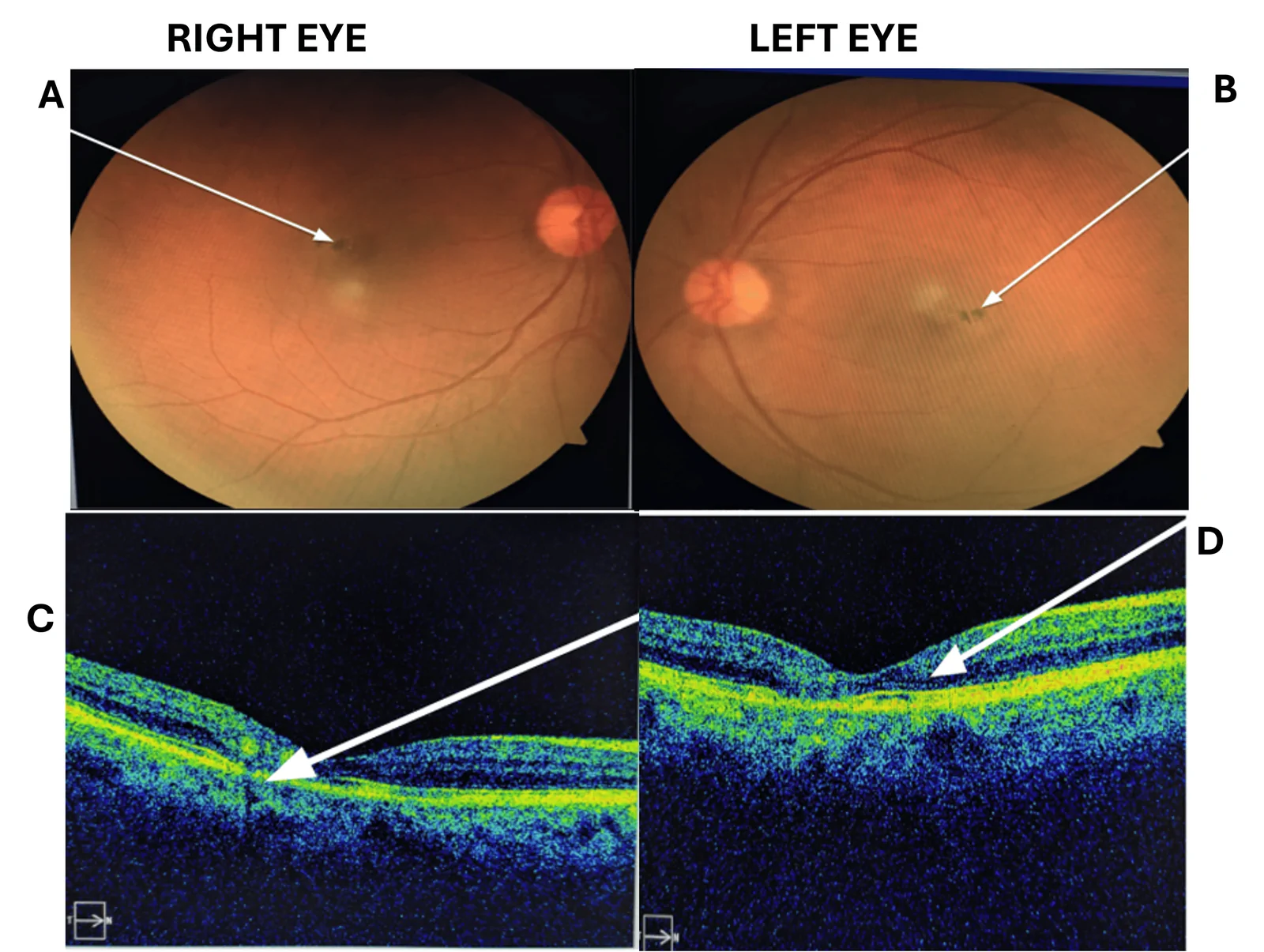
Fundus and optical coherence tomography (OCT) findings in Case B (A) Fundus photograph of the right eye reveals modest parafoveal retinal graying with loss of the typical foveal reflex (arrow). (B) Fundus photograph of the left eye shows mild parafoveal retinal graying and loss of the usual foveal reflex (arrow). (C) Right-eye OCT shows hyperreflectivity with shadowing at the fovea (arrow). (D) Left-eye OCT demonstrates cavitation secondary to lack of distinction of the IS/OS zone (arrow).

Case C 

A 52-year-old female without any known comorbidities had a BCVA of 6/24 in both eyes. Fundus examination showed modest bilateral parafoveal retinal graying with loss of the normal foveal reflex. OCT showed hyperreflectivity of the outer retinal layers with shadowing in both eyes. Figure 3 shows the corresponding fundus and OCT findings.

**Figure 3:**
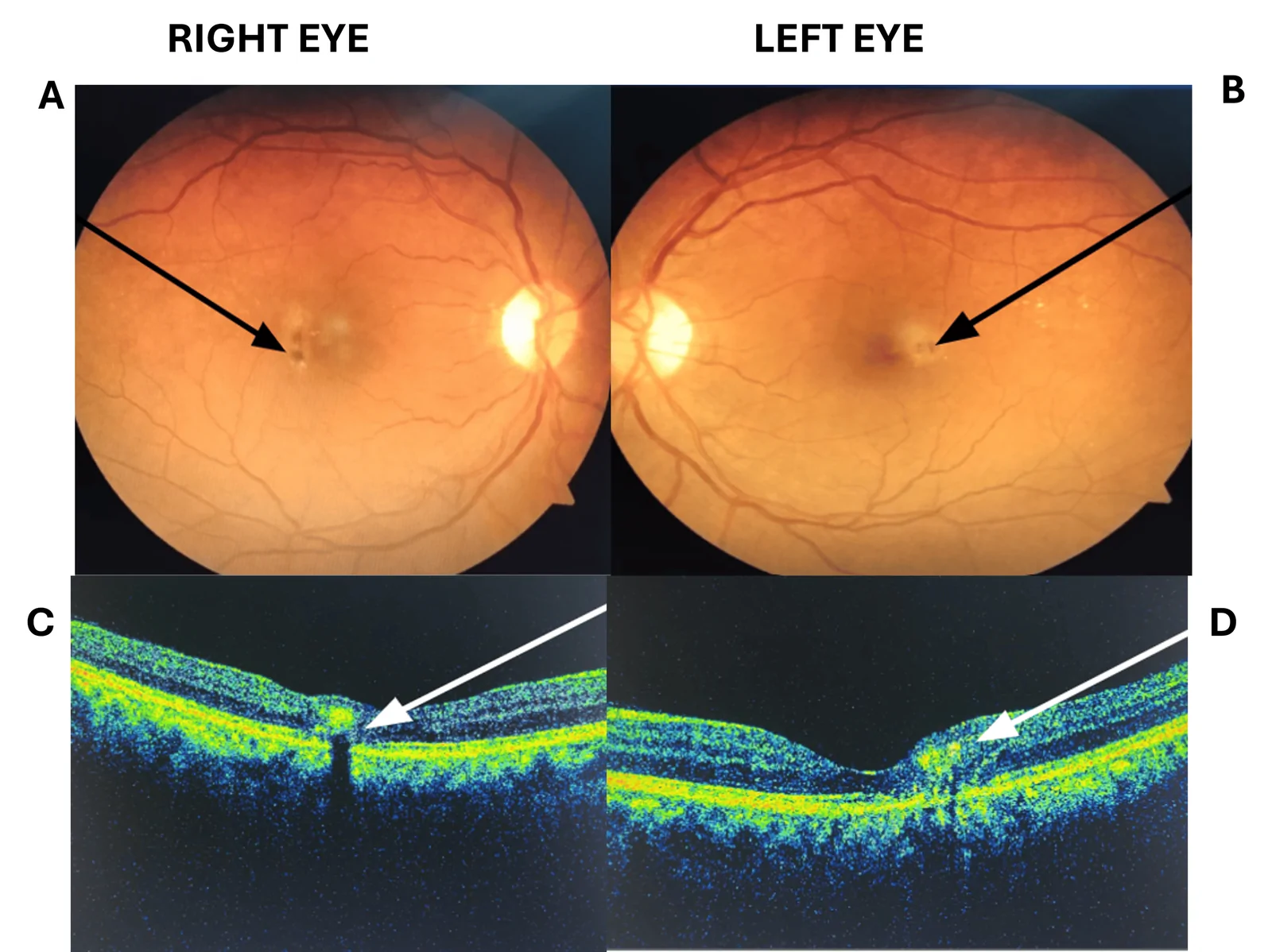
Fundus and optical coherence tomography (OCT) findings in Case C (A) Fundus photograph of the right eye demonstrating parafoveal retinal graying with loss of the typical foveal reflex. (B) Left-eye fundus photograph demonstrating comparable parafoveal retinal graying with loss of the normal foveal reflex. (C) Right-eye OCT revealing hyperreflectivity in the outer retinal layers with posterior shadowing (arrow). (D) Left-eye OCT demonstrating hyperreflectivity involving the outer retinal layers with posterior shadowing (arrow).

Case D

A 58-year-old female with no known comorbidities had a BCVA of 6/18 in the right eye and 6/36 in the left eye. Fundus examination revealed bilateral subtle parafoveal retinal graying with alteration of the normal foveal reflex. OCT demonstrated hyperreflectivity in the outer retinal layers with shadowing in both eyes. Figure 4 shows the corresponding fundus and OCT findings.

**Figure 4:**
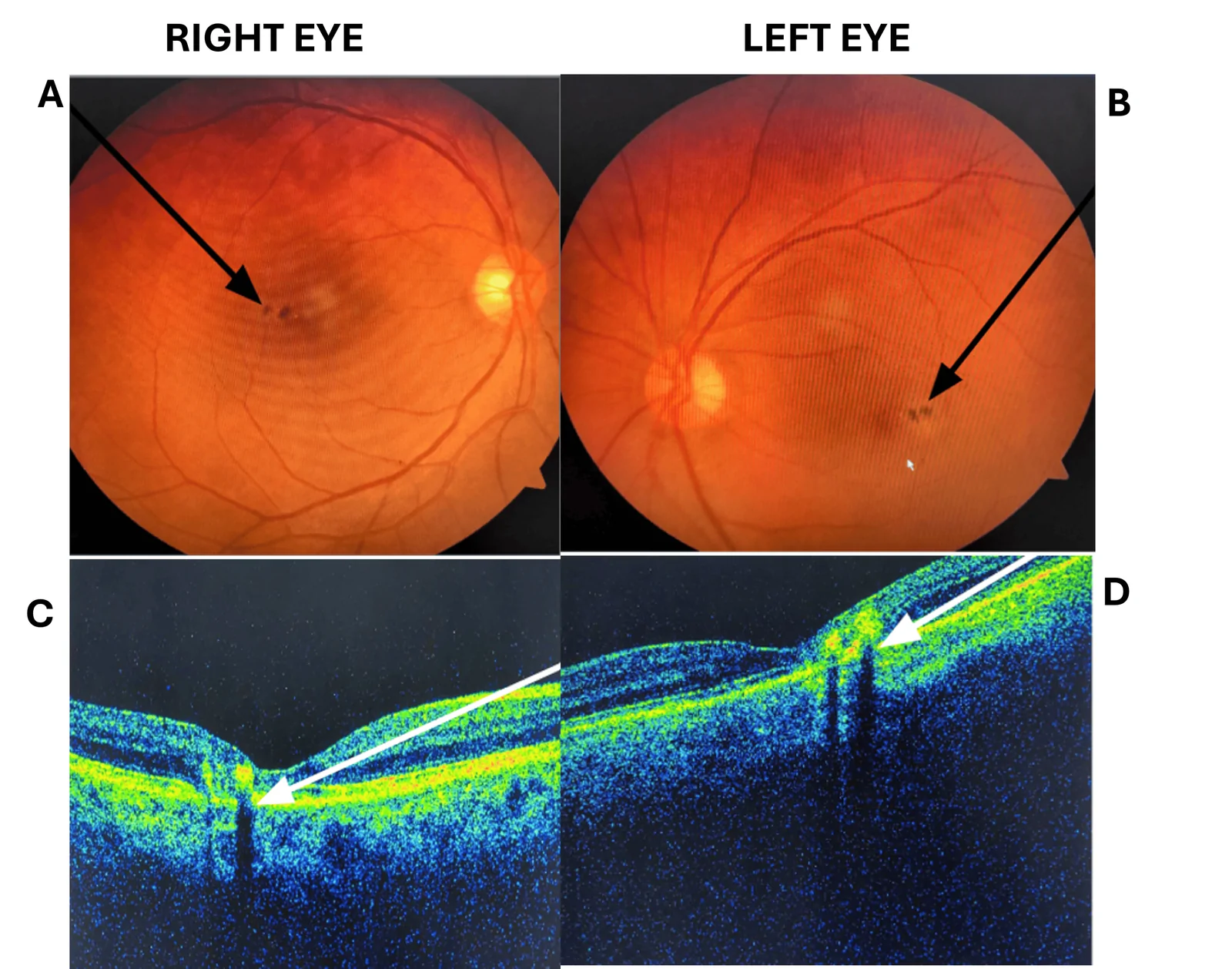
Fundus and optical coherence tomography (OCT) findings in Case D (A) Right-eye fundus photograph demonstrating parafoveal retinal graying with distortion of the usual foveal reflex. (B) Fundus photograph of the left eye demonstrating comparable parafoveal retinal graying with loss of the usual foveal reflex. (C) OCT of the right eye showing hyperreflectivity of the outer retinal layers with posterior shadowing (arrow). (D) Left-eye OCT demonstrating hyperreflectivity involving the outer retinal layers with posterior shadowing (arrow).

Case E 

A 55-year-old female with no known comorbidities presented with a BCVA of 6/36 in the right eye and 6/18 in the left eye. Fundus examination demonstrated modest bilateral parafoveal retinal graying with loss of the normal foveal reflex. OCT revealed hyperreflectivity of the outer retinal layers with shadowing in both eyes. Figure 5 shows the corresponding fundus and OCT findings.

**Figure 5:**
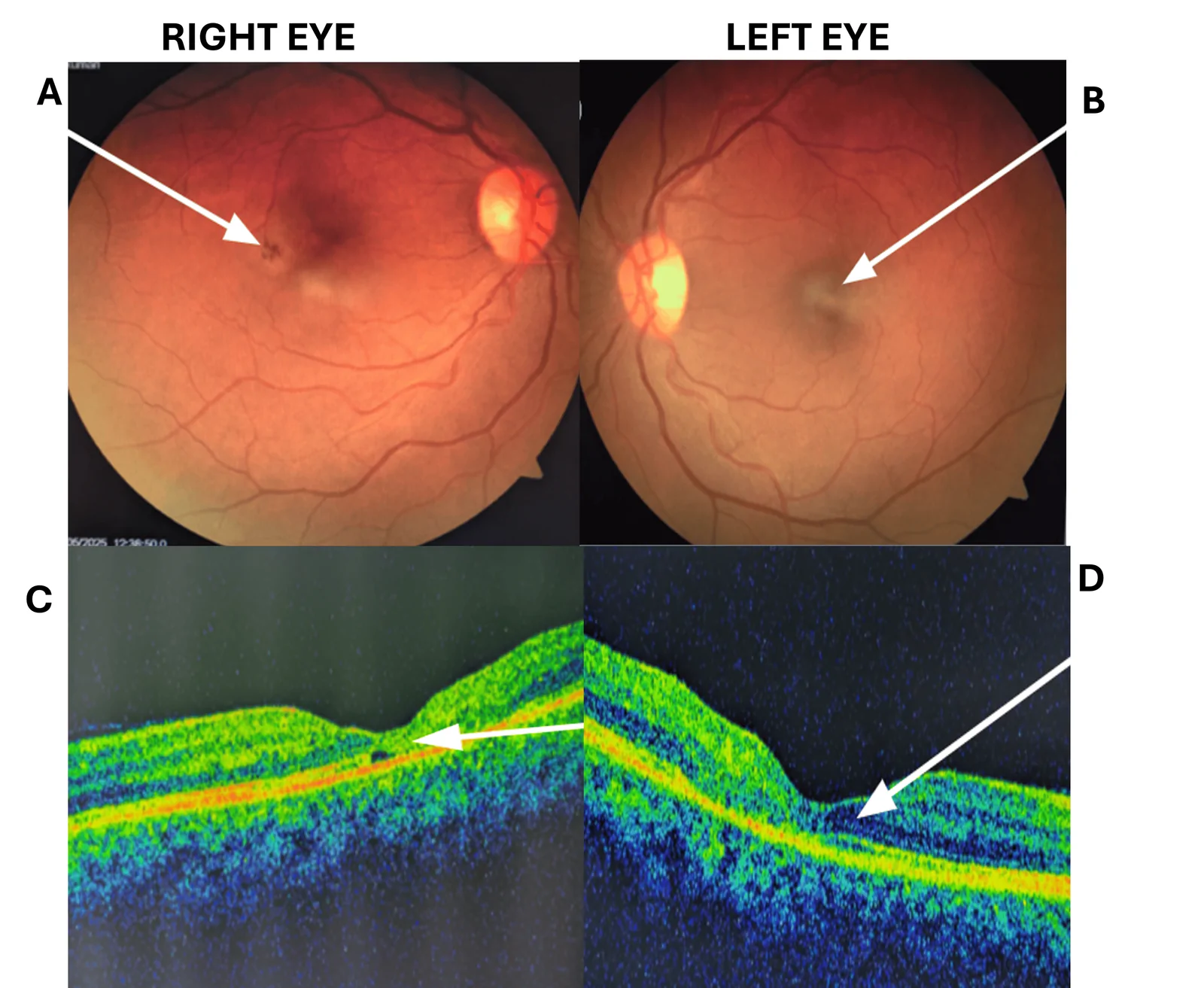
Fundus and optical coherence tomography (OCT) findings in Case E (A) Fundus photograph of the right eye demonstrating mild parafoveal retinal graying with loss of the usual foveal reflex (arrow). (B) Left-eye fundus photograph demonstrating comparable parafoveal retinal graying with loss of the normal foveal reflex (arrow). (C) Right-eye OCT demonstrating loss of differentiation of the IS/OS (ellipsoid) zone at the fovea (arrow). (D) Left-eye OCT demonstrating loss of differentiation of the IS/OS (ellipsoid) zone at the fovea (arrow).

Across the five patients, all demonstrated bilateral parafoveal retinal graying with alteration or loss of the normal foveal reflex on fundus examination. OCT demonstrated outer retinal hyperreflectivity with posterior shadowing in three patients, while loss of IS/OS differentiation was observed in the remaining patients; inner retinal cavitation was noted in one eye of one patient.

The fundus photographs and OCT images were reviewed by multiple authors of this article for the characteristic clinical and imaging features of MacTel type 2.

## Discussion

MacTel type 2 is an uncommon, progressive, bilateral macular disorder, historically described as idiopathic juxtafoveolar retinal telangiectasia, characterized by a distinctive combination of parafoveal vascular abnormalities and neuroretinal degeneration. Evidence to date supports the idea that MacTel type 2 is a complex neurodegenerative and metabolic retinal illness in which Müller cell abnormalities, photoreceptor dysfunction, and retinal metabolism are important. Vascular remodeling is an associated component of the disease process [1,2].

The current case series describes five patients with clinical and anatomical features consistent with MacTel type 2. The patients were mostly female (4/5) and older than 50 years, in accordance with the MacTel type 2 literature.

OCT is an important structural indicator in MacTel type 2 and was essential for the characterization of the present cases. The structural findings in the five individuals are consistent with the reported OCT findings of MacTel type 2. OCT has become an important structural biomarker for MacTel type 2, allowing extensive evaluation of the integrity of the retinal layers and providing objective evidence of the neurodegenerative component of the disease [1-4].

The OCT results described in the current series included foveal hyperreflectivity with posterior shadowing, loss of IS/OS or ellipsoid zone (EZ) differentiation, retinal cavitation, changes in the foveal pit, and loss of differentiation of the outer retinal and outer nuclear layers [1-4]. These changes are directly relevant to the current understanding of MacTel, as they represent indicators of retinal tissue degeneration and not just vascular leakage.

Hyper-reflection, foveal pit, and retinal cavitations

Multiple eyes demonstrated foveal hyperreflectivity with posterior shadowing, representing a hyperreflective alteration of the outer retinal layers and attenuation of the underlying OCT signal.

In one case, OCT revealed a foveal pit configuration with loss of IS/OS differentiation.

One eye had an intraretinal hyporeflective space with loss of IS/OS distinction, described as retinal cavitation. Thus, the findings in the present series suggest that OCT may be a useful structural biomarker for MacTel type 2.

The literature has reported associations between MacTel type 2, DM, and HTN [5]. In the present series, none of the five patients had recorded systemic comorbidities. This finding suggests the presence of MacTel type 2 in the absence of conventional vascular risk factors and underlines its complex genetic and metabolic pathophysiology [5,6].

Progression of illness

MacTel type 2 is a degenerative retinal disease, and long-term observations have indicated permanent structural changes and progression of retinal defects over time [7]. Serial OCT examination may thus be beneficial in recording progression, in particular by monitoring changes in the degree of EZ rupture and outer retinal loss.

Müller cell involvement in type 2 MacTel

Müller cells play an essential role in sustaining retinal homeostasis and supporting neuronal and vascular function. Müller cell dysfunction has been suggested to be a significant component of the neurodegenerative process in MacTel type 2. Changes in Müller cells may influence retinal metabolism and subsequent degeneration of the outer retinal layers and photoreceptors. This may explain some of the structural anomalies detected on OCT, including EZ disruption, retinal thinning, and intraretinal cavitation [8].

Genetic and metabolic pathways

Genetic and metabolic factors are increasingly implicated in the etiology of MacTel type 2. Genetic alterations in serine biosynthesis have been recognized as significant factors in disease pathogenesis and progression [9]. Genome-wide analyses have identified common genetic variations associated with susceptibility to MacTel type 2 [10]. These findings are consistent with the notion that MacTel type 2 is a complex genetic and metabolic neurodegenerative retinal disorder.

Middle-aged individuals with bilateral macular abnormalities should be evaluated for MacTel type 2, particularly if OCT demonstrates typical structural alterations. OCT is an important structural biomarker for detecting anomalies of the EZ, outer retina, and fovea that may precede substantial visual loss. The identification of cavitation with accompanying disruption of the EZ and retinal thinning may help differentiate MacTel from ordinary cystoid macular edema.

Strengths and limitations

The major strengths of this series are bilateral involvement in all five patients, female predominance (4/5), and the demonstration of multiple characteristic OCT abnormalities, including EZ disruption, retinal cavitation, and outer retinal changes. It was also interesting to note the absence of documented systemic comorbidities [5].

Limitations include a small sample size, cross-sectional design, and lack of longitudinal assessment. Such results are descriptive and cannot be generalized to the wider MacTel population. Detailed functional, genetic, and metabolic assessments were also lacking, limiting evaluation of the link between structural findings and underlying disease mechanisms. Future studies should include structural, vascular, functional, and molecular evaluations.

## Conclusions

In this five-case series, the clinical and OCT features of MacTel type 2 in middle-aged patients, with bilateral involvement in all cases and a female predominance of 80%, have been described. OCT offers a structural evaluation of the neuroretinal component of MacTel and is therefore an important biomarker of disease involvement and progression. Recognition of the characteristic OCT phenotype is therefore important for correct diagnosis and appropriate clinical management. It is important to distinguish between structural cavitation associated with MacTel and conventional exudative macular edema to avoid inappropriate treatment.
